# Mesenchymal Stem/Stromal Cell-Derived Extracellular Vesicles and Their Potential as Novel Immunomodulatory Therapeutic Agents

**DOI:** 10.3390/ijms18071450

**Published:** 2017-07-06

**Authors:** Verena Börger, Michel Bremer, Rita Ferrer-Tur, Lena Gockeln, Oumaima Stambouli, Amina Becic, Bernd Giebel

**Affiliations:** Institute for Transfusion Medicine, University Hospital Essen, University Duisburg-Essen, Virchowstr. 179, 45147 Essen, Germany; michel.bremer@uk-essen.de (M.B.); rita.ferrertur@uk-essen.de (R.F.-T.); lena.gockeln@gmx.de (L.G.); Oumaima.Stambouli@ruhr-uni-bochum.de (O.S.); Amina.Becic@ruhr-uni-bochum.de (A.B.)

**Keywords:** mesenchymal stem/stromal cells, cell therapy, extracellular vesicles, exosomes, microvesicles, immunomodulation

## Abstract

Extracellular vesicles (EVs), such as exosomes and microvesicles, have been identified as mediators of a newly-discovered intercellular communication system. They are essential signaling mediators in various physiological and pathophysiological processes. Depending on their origin, they fulfill different functions. EVs of mesenchymal stem/stromal cells (MSCs) have been found to promote comparable therapeutic activities as MSCs themselves. In a variety of in vivo models, it has been observed that they suppress pro-inflammatory processes and reduce oxidative stress and fibrosis. By switching pro-inflammatory into tolerogenic immune responses, MSC-EVs very likely promote tissue regeneration by creating a pro-regenerative environment allowing endogenous stem and progenitor cells to successfully repair affected tissues. Accordingly, MSC-EVs provide a novel, very promising therapeutic agent, which has already been successfully applied to humans. However, the MSC-EV production process has not been standardized, yet. Indeed, a collection of different protocols has been used for the MSC-EV production, characterization and application. By focusing on kidney, heart, liver and brain injuries, we have reviewed the major outcomes of published MSC-EV in vivo studies.

## 1. Mesenchymal Stem/Stromal Cells in Cell and Immune Therapies

Mesenchymal stem/stromal cells (MSCs) are fibroblastoid progenitor cells, which can be raised from different tissues, e.g., bone marrow, adipose tissue and umbilical cord. Typically, in vitro, they are expanded as plastic adherent cells. Propagated MSCs contain the ability to differentiate into various cell types, such as cells of the adipogenic, chondrogenic and osteogenic lineages [1,2,3]. The discovery that MSCs do not express Human Leuckocyte Antigen (HLA) class II encoded antigens led to the assumption that MSCs confer only low immunogenicity if transplanted in an allogeneic setting [4,5,6]. Upon studying the MSCs’ interaction with non-related immune cells, it turned out that, in addition to their low immunogenicity, MSCs are able to suppress the function of various immune effector cell types and to promote regulatory immune functions [5,7,8]. According to these features, MSCs became a very attractive cell source in regenerative medicine and immune therapy. So far, more than 800 clinical trials have been registered at the National Institute of Health (clinicaltrials.gov) aiming to use MSCs as novel therapeutic agents to treat an increasing number of different diseases. A large percentage of the registered clinical trials is destined to treat pathological conditions resulting in tissue loss, such as ischemic stroke and myocardial infarction, and to diseases caused by non-controllable pro-inflammatory responses, such as the steroid refractory acute graft-versus-host disease (aGvHD) or Morbus Crohn [9].

## 2. MSCs Exert Therapeutic Functions in a Paracrine Manner

Initially, MSCs were thought to exert their effects according to their multipotent differentiation capacity and by direct intercellular interactions, mainly with immune cells [5,7,8]. However, recent data imply that MSCs mediate their therapeutic functions in a paracrine rather than a cellular manner. In several studies, it was observed that following systemic administration, the engraftment of MSCs in damaged tissues was rather low; instead, most intravenously-applied MSCs were recovered in lungs and liver of treated subjects [10,11].

To best of our knowledge, Gnecchi and colleagues were the first who showed in the example of a rat ischemic heart model that the effects of MSCs were mediated in a paracrine manner [12]. Injection of conditioned media (CM) from MSCs overexpressing the gene *Akt1* was sufficient to reduce acute myocardial infarction (AMI) sizes and to improve ventricular functions [12,13]. Similarly, in a porcine model for AMI, intravenous and intracoronary injections of MSC-CM significantly improved the symptoms. Mechanistically, nuclear oxidative stress and apoptosis rates were reduced, which correlated with a reduction of infarction sizes and marked improvements of systolic and diastolic cardiac performances [14]. Pointing towards a cytokine mediated effect, Lee and colleagues showed in a mouse model for AMI that intravenously administered bone marrow-derived MSCs (BM-MSC) mainly got trapped in the lungs of treated animals [10]. Proposing a paracrine mode of action, the authors have started to search for cytokines being involved in this process. As a candidate, they identified the cytokine TSG-6, which in its recombinant form was able to resemble parts of the MSCs’ therapeutic effects. Underlining the importance of TSG-6 in this model, the siRNA mediated knockdown of TSG-6 expression was found to abrogate the therapeutic potential of corresponding MSCs completely [10].

In the example of an acute kidney injury (AKI) model, Tögel and colleagues showed that, although injected BM-MSCs transiently engrafted into damaged renal tissue, their beneficial effects on renal function and tubular damage were mediated by anti-apoptotic, promitogenic and vasculotropic factors. Notably, fibroblasts, which were applied as the control, failed to improve the symptoms [15,16]. Supporting the notion that MSCs mainly act in a paracrine manner as in AMI models, CM from BM-MSC were able to improve the kidney function in AKI rats [17]. The observation that CM from MSCs, but not that from mouse lung fibroblasts, can suppress hypoxia-induced pulmonary injury in mice indicates the existence of MSC-specific paracrine components. Since application of MSC-CM specifically blocked the invasion of macrophages in the injured lungs, a link to the immunomodulatory activities of MSC-CM was provided [18]. The observation that encapsulated BM-MSCs increased the survival rates and clinical score of GvHD mice to the same extent as systemically-administered MSCs provided further evidence that MSCs exert at least parts of their therapeutic functions by the release of immunomodulatory factors [19].

## 3. MSCs Exert Their Therapeutic Effects via Microvesicles and Exosomes

Becoming aware that MSCs act in a paracrine rather than a cellular manner, several groups started to search for the therapeutically-active components. Within the two landmark studies in the field, MSC-CM were fractioned by applying different protocols. Bruno and colleagues fractioned MSC-CM by ultracentrifugation and recovered the MSCs’ activity that suppressed murine acute tubular injury within the 100,000× *g* pellet. Upon characterizing the pellet, vesicular structures with sizes between 80 nm and 1 µm (mean value of 135 nm) were discovered, which were deciphered as microvesicles [20]. Similar to the in vivo observed effects of MSCs, the microvesicle fraction suppressed apoptosis rates and increased the proliferation of tubular epithelial cells in vitro to a similar extent as the MSCs themselves. Lai and colleagues used an HPLC-driven size-exclusion method and enriched a fraction containing particles with a hydrodynamic radius of 55–65 nm [21]. Due to the presence of the exosomal marker proteins such as CD9, CD81 and Alix, the authors used the term exosomes for the recovered particles. Upon testing the obtained exosome fraction in a murine model for AMI, a reduction of the infarction size was observed, which resembled the effects the group had already observed for MSCs and MSC-CM in a previous study [14].

## 4. Extracellular Vesicles

Cells can release a number of different membrane-surrounded vesicles of sizes ranging from a few nanometers to several microns into their extracellular environment. Collectively, these vesicles are named extracellular vesicles [22,23]. Historically, the term exosomes was initially used in the vesicle field for intraluminal vesicles (ILVs), which were found to be released into the extracellular environment upon fusion of late endosomes, the multivesicular bodies (MVBs), with the plasma membrane [24]. Due to the controlled assembly of the ILVs by the endosomal sorting complex required for transport (ESCRT) machinery, ILVs are comparable in size [25]. Depending on the techniques used, the sizes of the ILVs, which are released into the extracellular environment, vary between 70–100 nm (when analyzed by Transmission Electron Microscopy, TEM) and 120–150 nm (when analyzed by Nanoparticle Tracking Analysis, NTA) [26]. Although excreted ILVs can be enriched by different methods [27], the resulting fractions regularly also contain vesicles of similar sizes, which do not derive from the endosomal compartment. Initially, all nanosized extracellular vesicles (EVs) were named exosomes; however, to be more precise, members of the International Society of Extracellular Vesicles (ISEV) agreed to specifically use the term exosomes for ILV-corresponding vesicles. Nowadays, vesicles that bud off the plasma membrane are named microvesicles; they can be larger than exosomes and typically have said sizes of 100–1000 nm. A third class of very prominent vesicles arises when apoptotic cells get fragmented. According to the literature, apoptotic cells form apoptotic bodies with said sizes of 500 nm to several microns [22,23]. However, as commonly not mentioned in the literature, apoptotic cells also form vesicles in the same size range as exosomes and microvesicles. In addition, there are plenty of other sources of membrane-surrounded vesicles with a huge collection of different names, e.g., ectosomes, oncosomes, microparticles, etc. [28]. Due to the fact that no specific exosome and microvesicle markers have been identified yet and, for now, vesicles can only be fractioned according to their sizes and/or densities, but not regarding their origin, representatives of ISEV agreed on naming all experimentally-obtained vesicles as extracellular vesicles (EVs) [22,23,29].

As initially there were no agreed methods to characterize prepared EV fractions, the ISEV published a position paper defining some minimal criteria recommended for the characterization of purified EVs [30]. This includes semi-quantitative analysis of the EVs’ protein composition, commonly Western blots (WBs) for typical EV marker proteins, such as CD9, CD63, CD81, Alix or TSG101, size analysis by NTA, dynamic light scattering (DLS) or recessive pulse sensing (RPS) and analysis of their morphology regularly by TEM [30].

Starting with the discovery in 1996 that EVs released by B cells can promote T cell responses [31], it became evident that EVs constitute essential components of a newly-discovered intercellular communication system. [32]. Meanwhile, EVs have been harvested from all body fluids and were found to essentially take part in many physiological and pathophysiological processes [33]. Depending on their origin, EVs exert different functions. Maybe based on the landmark paper of Raposo and colleagues [31], the EV-mediated communication has best been investigated between tumor and immune cells and among different immune cell types. For example, mature dendritic cells have been found to release EVs promoting pro-inflammatory functions [34], while many tumor cells release EVs with anti-inflammatory and tolerance-inducing functions [32]. At the molecular level, the EVs’ functional properties are reflected to the presence of specific combinations of molecules, typically mirroring unique characteristics of their cells of origin. According to these characteristics, EVs have been recognized as a novel class of biomarkers for a variety of different diseases, which can often be detected in liquid biopsies from early disease stages on [35]. In this context, especially the discovery that EVs carry RNAs, which can effectively modulate gene expression in the EVs’ target cells, has promoted the field intensively [36,37,38].

## 5. MSC-EVs Exert Therapeutic Functions in Different Disease Models

Since the original description of the therapeutic potential of MSC-EVs in the AKI and MI models in 2009 and 2010 [20,21], respectively, approximately 80 original manuscripts have been published addressing the therapeutic functions of MSC-EVs in animal models. Up to now, the most addressed topics were heart, kidney, liver and brain injuries. Within the following part, this review gives a global overview of studies that have applied MSC-EVs to any of these injury models and have investigated the impacts of the applied MSC-EVs on immunobiological processes in vivo. Although our intention was to include all publications fulfilling these criteria, we would like to apologize in case we have missed any publication of relevance. Before discussing the MSC-EVs’ therapeutic properties, some basic features of the studies should be compared.

## 6. MSC-EV Production Strategies

Comparable to the MSC field, the MSCs for the EV production were obtained from different tissues and raised under different culture conditions. Some groups used serum- or human platelet lysate (PL)-supplemented media, while others used serum-free media (Table 1). Moreover, the preparation of the CM for the EV isolation was also different. Most of the groups used special EV-depleted media, while others used normal expansion media (Table 1). A high variability is also reflected by the EV-isolation methods, which had been used to isolate the EVs for the functional studies, ranging from ultracentrifugation- to chromatography-based methods (Table 1). Furthermore, the obtained EVs had been characterized in variable manners. It is not our intention to discuss the different parameters, here; however, to highlight the high variability in the MSC-EV production and characterization, we have included the information in Table 1.

## 7. Application of MSC-EVs in Animal Models

**Figure 1:**
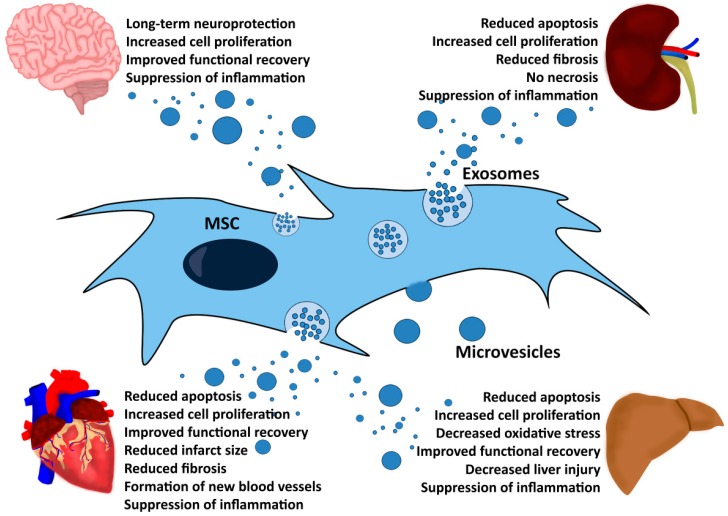
Overview of the MSC-EV-mediated therapeutic effects observed in animal models for kidney, heart, liver and brain injuries. MSC, mesenchymal stem cells, EV, extracellular vesicles.

Comparable to the MSC-EV production strategies and besides the fact that different animal species and strains were used as model systems, the way the in vivo studies were designed varies tremendously (Table 2). Most groups applied the MSC-EVs intravenously or intraperitoneally. However, also other application strategies were used, e.g., directly into injured tissues. Some groups applied the MSC-EVs once; while others used varying numbers of repetitive applications (up to five times; Table 2). Moreover, most of the publications deciphered the amount of the applied MSC-EVs regarding their protein concentration, which was very variable between studies. A few groups used particle numbers or cell equivalent doses to decipher their applied MSC-EV amounts. As diversely as the studies had been designed were the methods with which biological effects were characterized. A few studies searched for individual molecules that contributed to the observed effects. Several studies identified specific RNAs (mRNA and/or miRNA) as essential functional components of their applied MSC-EV fractions. However, no specific RNA or any other molecule was identified in several studies (Table 2). Remarkably, despite the high variability in the study designs, all studies observed improvements of the investigated disease/injury symptoms (Figure 1).

With respect to kidney diseases, mainly the effects of MSC-EVs on AKI were investigated. MSC-EV treatment was found to improve kidney function in these AKI models [39,40]. Specifically, MSC-EV administration was repetitively found to decrease AKI-induced oxidative stress, apoptosis and fibrosis [17,20,39,40,41,42,43,44]. Instead, MSC-EV treatment promotes angiogenesis and expansion of endogenous renal cells [17,20,41,42,43,45]. At the immunobiological level, MSC-EV treatment led to a reduction of pro-inflammatory and an increase of anti-inflammatory cytokines [17,44,46]. Related to this, AKI-induced invasion of macrophages and lymphocytes was suppressed in MSC-EV-treated AKI animals [39,43,47].

Regarding the heart, the MSC-EV therapeutic impacts were mainly studied in AMI models. Several studies observed that MSC-EV treatment in AMI models led to a reduction of infarction sizes and improvement of general heart functions [21,29,48,49,50,51,52,53,54]. Comparable to the AKI models, MSC-EV treatment reduced fibrosis and apoptosis, but promoted angiogenesis instead [29,48,49,50,51,53,54]. Furthermore, MSC-EV treatment was found to reduce the invasion of macrophages and eventually other immune cells into the affected heart regions [49,50].

The MSC-EVs’ impact on liver diseases was studied in models for acute liver injury (ALI), hepatic failure and hepatic ischemia/reperfusion injury. MSC-EV treatment improved liver functions in all models and induced anti-apoptotic effects [55,56,57,58,59]. Like in the previous models, MSC-EV administration resulted in a decline of pro-inflammatory reactions including immune cell invasion and oxidative stress [55,56,57,59,60].

Regarding the brain, impacts of MSC-EV treatment were mainly studied in models for ischemic stroke and traumatic brain injury (TBI) [61,62,63,64,65,66,67,68,69,70,71]. In addition, we studied the therapeutic effects of MSC-EVs in a rat model for inflammation-induced preterm and a sheep model for hypoxia-induced fetal brain injury [72,73]. Almost all studies that used naive MSC-EVs showed an MSC-EV-mediated improvement of cognitive deficits or function, respectively [62,68,69,71,72,73]. Coupled to the functional recovery, more neural cells were generated in MSC-EV-treated than in control animals [62,63,68]. Comparable to the other organ systems discussed before, MSC-EV treatment reduced apoptosis rates in affected brains, but promoted angiogenesis and neurogenesis instead [63,66,70,71,73,74]. Both, systemic pro-inflammatory and neuro-inflammatory cues were reduced following MSC-EV treatment. Amongst others, the number of invading macrophages into the affected brain areas was found to be reduced [62,63,64,68,70,72,73].

## 8. MSC-EVs in the Clinics

So far, two studies have been published in which MSC-EVs were applied to human patients. In the first study [75], MSC-EVs were administered in an allogeneic setting to a patient suffering from steroid refractory graft-versus-host disease (GvHD). Upon applying MSC-EVs in escalating doses, GvHD symptoms declined long term, and steroid doses could be reduced. Upon analyzing the immunomodulatory activity of the applied MSC-EV fraction in a mixed lymphocyte reaction (MLR) assay before MSC-EV treatment, MSC-EVs were able to suppress the number patient-derived peripheral blood cells, which secreted the pro-inflammatory cytokines IL-1β, TNFα and IFNγ. During the course of the treatment and in the absence of any additionally in vitro applied MSC-EVs, the number of patient-derived peripheral blood cells, which secreted IL-1β, TNFα and IFNγ, within the MLR assays declined over time. Since these data reflected the clinical GvHD symptoms, the data suggest that MSC-EVs can modulate the status of the patients’ immune cells in a sustained manner. The applied MSC-EV fraction was shown to contain the anti-inflammatory cytokines TGF-β, IL-10 and HLA-G. Notably, by comparing the concentration of these cytokines in four independent MSC-EV fractions, higher levels were found in the MSC-EV fraction that was applied to the patient than in the three remaining ones. To this end, it has not been investigated whether the other MSC-EV fractions have lower immunomodulatory capabilities than the applied MSC-EV fraction. It remains an open question whether the MSC-EVs’ anti-inflammatory capabilities were associated with their relatively high TGF-β, IL-10 and HLA-G levels, or whether other EV components controlled the improvement of the GvHD symptoms. However, for a time interval of more than four months, the MSC-EV therapy resulted in significant improvement of clinical GvHD symptoms of the treated patient [75].

The second study addressed the therapeutic impact of MSC-EVs in patients with chronic kidney disease (CKD) [76]. Forty patients were included in this study. Half of them were placebo treated, the other half with MSC-EVs. MSC-EVs were applied twice, for the first treatment intravenously and, for the second treatment, one week later, intra-arterial. Without showing any side effects and in contrast to the control group, the MSC-EV-treated group showed significant improvements of the kidney function as measured by a variety of different markers, i.e., the estimated glomerular filtration rates (eGFR), the urinary albumin to creatinine ratio and the blood urea and serum creatinine levels. Impacts on the immune system were studied by analyzing TGF-β, IL-10 and TNFα concentration in the peripheral blood. TGF-β and IL-10 concentrations were increased massively in MSC-EV-treated patients short term (12 weeks) and were even detected in elevated levels one year after MSC-EV treatment. In contrast, the pro-inflammatory cytokine TNFα was decreased in the MSC-EV-treated group shortly after MSC-EV administration and remained low during the following year of observation. Upon taking biopsies from the patients’ kidneys, an increased number of CD133/Ki67 tubular cells (putative cycling renal progenitor cells) was discovered in the biopsies of the MSC-EV-treated patients, but not in those of the control groups, suggesting that the MSC-EV therapy triggered the regeneration within the affected kidneys [76].

## 9. MSC-EVs as a Novel Therapeutic Agent

Despite the variability in the MSC-EV production and application, the results of the different animal models and the two clinical applications demonstrate positive therapeutic effects of MSC-EVs. So far, no side effects have been reported, implying that MSC-EVs application can in principle, be considered as safe. According to their therapeutic potential and a number of advantages over cellular therapeutics [77], several groups have started with efforts to translate MSC-EVs into the clinics. In this respect, a number of guidelines need to be fulfilled. As EVs are novel therapeutic agents, these guidelines have not been defined yet, but might at least partially be adopted from other guidelines for cellular therapeutics. A comprehensive overview about potential guidelines and recommendations for the production, quality assurance and application of EV-based therapeutics have recently been provided in an ISEV and European Network on Microvesicles and Exosomes in Health and Disease (ME-HaD) position paper [77].

## 10. Conclusions and Perspectives

Although MSC-EVs seem to exert positive impacts on tissue specific stem cells, promote angiogenesis and suppress oxidative stress and fibrosis (Figure 1), according to our understanding, their most important impact is to suppress pro-inflammatory responses in all disease models discussed. Supported by the findings in our ischemic stroke model that in an untreated situation, neural progenitors are not able to effectively create mature neural cells [63], we would like to speculate that pro-inflammatory environments are not permissive for endogenous stem and progenitor cells to initiate regenerative processes. To our understanding, endogenous stem and progenitor cells require a tolerogenic environment to survive and to successfully promote regeneration. Indeed, at the cellular level, it was shown, in vitro, that MSC-EVs are able to convert M1 into M2 macrophages and that EVs released by M2 macrophages can subsequently promote regulatory T-cell formation [78]. Thus, by switching pro-inflammatory into tolerogenic environments, MSC-EV administration might promote regenerative processes.

Coupled to the lack of standardization and the high variability in MSC expansion and EV purification protocols, it appears very likely that differences in experimental strategies to prepare MSC-EVs for the therapeutic setting will result in MSC-EV fractions showing different immunomodulatory properties. Furthermore, in our past and on-going work, we have experienced that, maybe attributed to the different presence of certain cytokines, MSC-EV preparations vary in their immunomodulatory activities, eventually in a donor-dependent manner [75]. Accordingly, it has to be considered that not all MSC-EV fractions provide sufficient therapeutic activities to improve the clinical symptoms of the disease to be treated. To identify MSC-EV fractions with the highest therapeutic potential, appropriate potency assays need be set up. However, to set up optimal potency assays, the mode of action of the MSC-EVs needs to be unraveled. Furthermore, it will be required to compare the in vitro activities of different MSC-EV fractions with their therapeutic potential in vivo. Considering that their effect is mainly mediated by tolerance-inducing activities, it will be interesting to learn whether MSC-EV fractions with high therapeutic potentials in one disease model will also have high therapeutic potentials in other diseases models or whether each disease model requires its own optimal MSC-EV fraction.

## Figures and Tables

**Table 1:** Experimental parameters of the MSC expansion and EV harvesting and characterization strategies reported for MSC-EV in vivo studies.

Ref.						EV Harvesting Conditioning															
	Disease	MSC Origin	Tissue Source	MSC Isolation	Supplement	Media	Time	EV Isolation	Pre-Processing	Filter	Final Purification Step	Characterization					Tested EV-Marker				
												NTA/DLS	Protein	TEM	FLOW	Other	CD9	CD63	CD81	TSG101	Other
[41]	AKI	Human	UC	unfractionated	10%	serum free	48 h	UF + sucrose + UC	1000× *g*, 2000× *g*, 10,000× *g*	100 kDa, 0.22 µm	100,000× *g*, 60 min										
[20]	AKI	Human	BM	Ficoll	10% FBS	0.5% BSA	o.n.	UC	2000× *g*		100,000× *g*, 2 × 60 min										
[40]	AKI	Human	BM	commercial	serum free	0.5% BSA	o.n.	UC	10,000× *g*		100,000× *g*, 60 min										
[17]	AKI	Rat	BM	unfractionated	20% FCS	EV depletion	48 h	UC	800× *g*, 2000× *g*,	0.1 µm	100,000× *g*, 2 × 60 min										
[42]	I/R AKI	Human	UC	unfractionated	10% FBS	0.5% BSA	o.n.	UC	2000× *g*		100,000× *g*, 60 min										
[43]	I/R AKI	Human	WJ	unfractionated	10% FBS	0.5% BSA	o.n.	UC	2000× *g*		100,000× *g*, 60 min										
[79]	I/R AKI	Human	UC	Ficoll	10% FBS	serum free	24 h/ 48 h	UC	2000× *g*		100,000× *g*, 60–120 min										
[45]	I/R AKI	Human	BC	unfractionated	n.d.	serum free	o.n.	UC + Optiprep	3000× *g*,		100,000× *g*, 120 min 350.000× *g*, 60 min 100.000× *g*, 60 min										
[44]	I/R AKI	Rat	AT	unfractionated	10% FBS	EV depletion	96 h	UC	4500 rpm	0.22 µm	120,000× *g*, 90 min										
[47]	I/R renal injury	Mouse	BM	commercial	10% FBS	EV depletion	48 h	UC		0.22 µm	n.d.										
[39]	Renal Injury	Mouse	BM	unfractionated	10% FBS	n.d.	n.d.	UC	2000× *g*,		100,000× *g*, 2 × 60 min										
[52]	Retinal Injury	Human	UC	unfractionated	10% FBS	serum free	n.d.	UC	200× *g*, 2000× *g*, 10,000× *g*		110,000× *g*, 120 min										
[46]	Renal Allograft	Rat	BM	unfractionated	20% FBS	EV depletion	16 h	UC	2000× *g*, 12,000× *g*		100,000× *g*, 70 min										
[76]	CKD	Human	UC	n.d.	serum free	0.5% HSA	o.n.	UC	2000× *g*		100,000× *g*, 60 min										
[29]	AMI	Human	BM	Ficoll	10% FCS	EV depletion	n.d.	UC	1500× *g*		100,000× *g*, 60 min										
[48]	AMI	Human	UC	unfractionated	10% FBS	EV depletion,	48 h	UC+ sucrose	300× *g*, 2000× *g*, 10,000× *g*,	100 kDa	100,000× *g*, 120 min										
[49]	AMI	Rat	BM	unfractionated	10% FBS	EV depletion	48 h	Precipitation (kit)	2000× *g*,		10,000× *g*, 60 min										
[50]	AMI	Rat	BM	n.d.	n.d.	EV depletion	48 h	Exoquick			1500× *g*, 30 min										
[51]	AMI	Human	UC	unfractionated	10% FBS	EV depletion	48 h	UF + sucrose + UC	300× *g*, 2000× *g*, 10,000× *g*,	100 kDa,	100,000× *g*, 120 min										
[54]	AMI	Rat	BM	unfractionated	15% FBS	EV depletion	48 h	Exoquick	3000× *g*	100 kDa	1500× *g*, 30 min										
[21]	AMI	Human	ESC	sorting of CD105+	10% FCS	n.d.	n.d.	TFF + filter steps		10, 1000, 500, 300 kDa	100 kDa										
[53]	AMI	Mouse	BM	unfractionated	serum free	serum free	n.d.	Exoquick	3000× *g*	0.3 µm	1500× *g*, 30 min										
[80]	AMI	Human	UC	unfractionated	10% FBS	EV depletion	48 h	UF + sucrose + UC	300× *g*, 2000× *g*, 10,000× *g*,	100 kDa	100,000× *g*, 120 min										
[81]	I/R injury	Human	ESC	sorting of CD105 +	n.d.	serum free	72 h	TFF + HPLC	500× *g*	0.22 µm, 100 kDa	Chromatography										
[58]	ALiI	Human	ESC	sorting of CD105+	10% FCS	serum free	72 h	TFF+HPLC		100 kDa	Chromatography										
[59]	ALiI	Human	UC	unfractionated	10% FCS	EV depletion	48 h	UC + Sucrose +UF	2.000× *g*,	100 kDa, 0.22 µm	100,000× *g*, 60 min										
[55]	Hepatic failure	Human	MB	Ficoll	20% FBS	20 % FBS	24 h	Exoquick	2000× *g*,	0.22 µm, 30 kDa	1500× *g*, 30 min										
[56]	Hepatic failure	Mouse Human	BM	commercial unfractionated	FBS	EV depletion	48 h	UC	300× *g*, 2000× *g*, 10,000× *g*,		100,000× *g*, 70 min										
[60]	Liver fibrosis	Human	UC	unfractionated	10% FBS	EV depletion	24 h	UF + Sucrose + UC	1000× *g*, 2000× *g*, 10,000× *g*		100,000× *g*, 60 min										
[57]	I/R injury	Human	iPSC	iPS derived	10% FBS	serum free	48 h	UF	300× *g*, 2000× *g*, 4000× *g*,	0.22 µm	Amicon Ultra 15										
[66]	Stroke	Rat	BM	unfractionated	20% FBS	EV depletion	24 h	UC	10,000× *g*	0.22 µm	100,000× *g*, 180 min										
[67]	Stroke	Rat	BM	unfractionated	20% FBS	EV depletion	24 h	UC + Sucrose	100,000× *g*	0.22 µm	100.000× *g*, 180 min										
[69]	Stroke	Rat	BM	unfractionated	20% FBS	EV depletion	24 h	UC	3000× *g*, 10,000× *g*	0.22 µm	100,000× *g*, 120 min										
[65]	Stroke	Rat	BM	unfractionated	20% FCS	EV depletion	24 h	UC	3000× *g*, 10,000× *g*	0.22 µm	100,000× *g*, 120 min										
[63]	Stroke	Human	BM	Ficoll	5% PL	5% PL	48 h	PEG + UC		0.22 µm	110,000× *g*, 2 h										
[64]	Stroke	mini-pigs	AT	unfractionated	10% FBS	EV depletion	96 h	UC	4500 rpm	0.22 µm	120,000× *g*, 90 min										
[62]	TBI	Human	BM	Ficoll	20% FBS	EV depletion	48 h	Exoquick			1500× *g*, 30 min										
[61]	TBI	Human	BM	unfractionated	17% FBS	serum free	6-48 h	UC	2565× *g*		100,000× *g*, 60–720 min										
[68]	TBI	Rat	BM	unfractionated	20% FBS	EV depletion	48 h	Exoquick			1500× *g*, 30 min										
[73]	Brain injury	Human	BM	Ficoll	10% PL	10% PL	48 h	PEG + UC	10,000× *g*	0.22 µm	110,000× *g*, 2 h										
[72]	Brain injury	Human	BM	unfractionated	10% PL	10% PL	48 h	PEG	10,000× *g*	0.22 µm	1500× *g*, 30 min										
[70]	Cerebral apoplexy	Human	BM	Ficoll	5% PL	culture media	48 h	PEG		0.22 µm	n.d.										
[71]	SCI	Rat	AT	digestion	n.d.	EV depletion	24 h	Kit (miRCURY)			3200× *g*, 30 min										
[75]	GvHD	Human	BM	unfractionated	5% PL	culture media	48 h	PEG + UC		0.22 µm	100,000× *g*, 120 min										
[82]	GvHD	Human	UC	unfractionated	serum free	serum free	48 h	UC	2000× *g*		100,000× *g*, 2 × 120 min										
[83]	Enterocolitis	Mouse	BM	unfractionated	10% FBS	serum free	48 h	Kit (P100 Pure Exo)													
[84]	Diabetes	Rat	BM	unfractionated	15% FBS	EV depletion	24 h	Precipitation (Kit)			10,000× *g*, 60 min										
[85]	Radiation damage	Human	BM	commercial	15% FBS	EV depletion	7 days	UC	300× *g*, 2000× *g*, 10,000× *g*,		100,000× *g*, 60 min										
[86]	Wound healing	Human	UC	unfractionated	serum free	serum free	48 h	UC + sucrose	1000× *g*, 2000× *g*, 10,000× *g*	100 kDa, 0.22 µm	100,000× *g*, 60 min										
[87]	Wound healing	Human	UC	unfractionated	10% FBS	n.d.	24 h	UC	10,000× *g*	0.22 µm	100,000× *g*, 180 min										
[88]	ALuI	Human	BM	n.d.	n.d.	0.5% HSA	48 h	UC	3000× *g*		100,000× *g*, 60 min										
[89]	ALuI	Human	BM	commercial	10% FCS	0.5% HSA	48 h	UC	10,000× *g*		100,000× *g*, 60 min										
[90]	Airway inflammation	Human	BM	n.d.	20% FBS	serum free	48 h	UC	3000× *g*		100,000× *g*, 2 × 60 min										
[78]	Graft rejection	Human	ESC	differentiation	serum free	serum free	72 h	TFF + HPLC		100 kDa	Chromatography										
[91]	Sepsis	Mouse	BM	unfractionated	15% FBS	EV depletion	24 h	UC	3000× *g*, 13,000× *g*	0.22 µm	36,000 rpm, 180 min										
[92]	Colitis	Rat	BM	unfractionated	10% FBS	serum free	48 h	UC	2000× *g*		100,000× *g*, 2 × 60 min										

EV, extracellular vesicle; AKI, acute kidney injury; I/R, ischemia/reperfusion; AMI, acute myocardial injury; ALiI, acute liver injury; TBI, traumatic brain injury; SCI, subortical Stroke; GvHD, graft-versus-host-disease; ALuI, acute lung injury; UC, umbilical cord; BM, bone marrow; WJ, Wharton jelly; BC, bowman’s capsule; AT, adipose tissue; ESC, embryonic stem cells; MB, menstrual blood; iPSC, induced pluripotent cells; n.d., not defined; FBS, fetal bovine serum; FCS, fetal calf serum; PL, platelet lysate; BSA, bovine serum albumin; HSA, human serum albumin; h, hours; o.n., over night; UF, ultrafiltration; TFF, tangential flow filtration; UC, Ultracentrifugation; HPLC, high-performance liquid chromatography; PEG, Polyethylene glycol; g, g-force; kDa, kilo Dalton; µm, micrometre; min, minutes; rpm, rounds per minute; NTA, nanoparticle tracking analysis; DLS, dynamic light scattering; TEM, transmission electron microscopy; FLOW, Flow cytometry; CD, cluster of differentiation.

**Table 2:** Application and analyses schemes of MSC-EV in vivo studies.

Organ	Reference	Disease	Animal			Xenogenic Application	Functional Testing In Vitro	Application	EV Dose	No. of Injections	Factors	Immunomodulatory Effects	Described Effects After MSC-EV Application
			Species	Strain	Gender								
Kidney	[41]	AKI	Rat	SD	f	y		Renal capsule	200 µg	1	Bcl-2, Bax		Reduced apoptosis Increased cell proliferation
	[20]	AKI	Mouse	SCID	m	y	EV uptake	i.v.	15 µg	1	mRNA dependent	RNA shuttled in MV associated with immune regulation	Morphological recovery Reduced apoptosis Increased cell proliferation
	[40]	AKI	Mouse	SCID	m	y	Apoptosis assay	i.v.	100 µg 100 µg + 50 µg	1 or 5	RNA dependent, ACTB, POLR2E SUMO-1		Improved survival Single injection: increased renal function, morphology and survival (although negative impact in the long-term) Multiple injections: decreased mortality (no impact in the long-term)
	[17]	AKI	Rat	Wistar	f	n		i.v.	100 µg/mL	1	mRNA dependent	Infiltrated lymphocytes T-B cell count higher, NK reduced TNFα transcripts reduced	Increased cell proliferation No necrosis
	[42]	I/R AKI	Rat	n.d.	m	y		i.v.	100 µg	1	RNA dependent, VEGF, HIF-1α		Reduced fibrosis Increased vessel density Reduced apoptosis Increased cell proliferation
	[43]	I/R AKI	Rat	SD	m	y		i.v.	100 µg	1	miR16, miR15b, miR15a	Reduced infiltration of macrophages (CD68 +)	Reduced apoptosis Increased cell proliferation
	[79]	I/R AKI	Rat	SD	m	y	T-reg induction T-cell proliferation	i.a.	n.d.	1	Apolipoprotein, galectins CD73, CD90		No necrosis No tubular dilation No cast formation
	[45]	I/R AKI	Mouse	SCID	m	y		i.v.	1 × 10^5^ CE	1	RNA dependent		Increased tubular cell proliferation
	[44]	I/R AKI	Rat	SD	m	n		i.v.	100 µg	1	NFĸB, IL-1ß, MIF, PAI-1, COX-2 re	Reduced inflammatory reaction reduced TNFα	Reduced oxidative stress Reduced mitochondrial damage
	[47]	I/R renal injury	Mouse	Balb/C	n.d.	n		Renal capsule	200 µg	1	CCR-2	Inhibition effect on recruitment of Monocytes and Macrophages	CCR2 enriched in Evs → binding to extracellular CCL-2
	[39]	Renal injury	Mouse	C57BL/6	n.d.	n		i.v.	30 µg	3		Lymphocyte infiltration	Improved renal function Decreased injury Prevented fibrosis
	[46]	Renal allograft	Rat	Lewis	m	n		n.d.	n.d.	1		Infiltrated lymphocytes T- and B-cell count higher, NK cells reduced TNFα transcripts reduced	no difference in kidney function
Heart	[29]	AMI	Rat	Wistar	m	y		Border zone heart	20 µL	4			Formation of new blood vessels Reduced infarct size
	[48]	AMI	Rat	SD	n.d.	y	Apoptosis assay	i.v.	400 µg	1	AKT overexpression, PDGF-D		Formation of new blood vessels Improved cardiac function
	[49]	AMI	Rat	SD	m	n		Infarct border	20 µg	1	miR29, miR24 upregulated miR34, miR130, miR378 downregulated	Reduced inflammation	No fibrosis Improved cardiac function Increased cell proliferation and migration
	[50]	AMI	Rat	SD	m	n	T-cell proliferation Tube formation EV uptake	Infarct border	80 µg	1		Decreased proliferation of inflammatory cells	Formation of new blood vessels Improved cardiac function
	[51]	AMI	Rat	SD	m	y	Tube formation EV uptake	i.v.	400 µg	1	Bcl2		Improved cardiac function Reduced fibrosis Increased cardiomyocyte proliferation
	[54]	AMI	Rat	SD	f	n		Intramyocardial	4 × 10^6^ CE	1	miR22, miR19, PTEN		Improved cardiac function Reduced infarct size Reduced apoptosis
	[21]	AMI	Mouse	n.d.	n.d.	y		i.v.	0.4 µg	1		Independent of immune cells	Reduced infarct size
	[53]	AMI	Mouse	C57BL/6	n.d.	n		Infarct border	1 µg	1	miR122		Reduced apoptosis Reduced fibrosis Improved cardiac function
Liver	[58]	Acute liver injury	Mouse	C57BL/6	m	y		i.s.	0.4 µg	1	HGF, HGFR protein, IL6ST/gp130, TNFRSF1A/TNFR1, CXCL2/MIP-2 protein, iNOS, NO, COX2, MIP-2		Decreased apoptosis Decreased liver injury Induced hepatocyte proliferation
	[59]	Acute liver injury	Mouse	BALB/c	n.d.	y		i.v. or oral	8/16/32 mg/kg BW	1	GPX1, Bcl2, ROS, MDA	Reduced serum levels of pro-inflammatory cytokines	Rescued liver failure Increased viability Decreased oxidative stress
	[55]	Hepatic failure	Mouse	C57BL/6	m	y	EV uptake Apoptosis assay	i.v.	1 µg/µL	1	Caspase-3, TNF-α, IL-6, IL-1ß	Inhibitory immunomodulation of activated MNCs decreased NK-cells	Reduced apoptosis Improved liver function
	[56]	Hepatic failure	Mouse	C57BL/6	m	Y *		i.p./i.v.	2 ×10^8^ to 2 × 10^10^ EVs	1	Y-RNA-1, MIP2, IL-6, IL-1 alpha, MIP-3 beta, IP-10, MCP-1, MCP-3		No apoptosis Reduced hepatic injury Improved survival
	[57]	Hepatic I/R injury	Rat	SD	m	y		i.v.	600 µg	1	TNF-α, IL-6, HMGB-1	Reduced inflammatory markers Reduced infiltration of inflammatory cells	Reduced necrosis/ apoptosis Decreased liver injury Decreased oxidative stress Induced hepatocyte proliferation
Brain	[66]	Stroke	Rat	Wistar	m	n		i.v.	100 µg	1			Improved neurological function Neurovascular remodeling
	[69]	Stroke	Rat	Wistar	m	n		n.d.	100 µg	1	miR-133		Improved functional recovery
	[65]	Stroke	Rat	Wistar	m	n		i.v.	100 µg	1	miR17-92 Cluster PTEN		Improved neurological function Increased neural remodeling
	[63]	Stroke	Mouse	C57BL/6	m	y		i.v.	2 × 10^6^ CE	3		Reduced T-call activation B-cell, NK-cell, T-cell lymphopenia	Long-term neuroprotection Increased angioneogenesis
	[64]	Stroke	Rat	n.d.	m	y		i.v.	100 µg	1	MMP-9, IL-1ß, TNFα, RANTES, PAI-1, NF-KB, iNOS, NOX-1, NOX-2, c-casp3, c-PARP p-SMAD3, TGF-ß, SMAD1/5, BMP-2	Reduced infiltration of CD11+ and CD68+cells	Decreased oxidative stress Increased angiogenesis
	[62]	TBI	Rat	Wistar	m	y		i.v.	100 µg	1		Reduced neuroinflammation reduced CD68+ cells at infarct zone	Improved functional recovery Increased cell proliferation Reduced neuroinflammation
	[68]	TBI	Rat	Wistar	m	n		i.v.	100 µg	1		Reduced neuroinflammation reduced CD68+ cells at infarct zone	Improved functional recovery Increased cell proliferation Reduced neuroinflammation
	[61]	TBI	Mouse	C57BL/6	m	y		i.v.	30 µg	1		Suppressing Neuroinflammation	Rescue cognitive impairments
	[73]	Brain injury	Rat	Wistar	n.d.	y		i.p.	1 × 10^8^ CE/kg BW	1		Modulated inflammatory responses	Improved cognitive function Reduced cellular degeneration
	[72]	Preterm brain injury	Sheep	Texel	n.d.	y		i.v.	2 × 10^7^ CE	2	IBA-1	Increased immunoreactivity	Decreased structural injury Functional neuroprotective effects Improved function
	[70]	Cerebral apoplexy	Rat	n.d.	n.d.	y		i.v.	2.4 × 10^4^ EVs	3		Reduced quantity of B-cells, NK cells, and T-cells all increased; neuroinflammation (fewer CD68+ cells in infarct zone) attenuated immunosuppression (reduced numbers of activated T-cells)	Identical effect of MSCs and MSC-EVs Increased neuron survival
	[71]	SCI	Rat	SD	male	n		i.v.	100 µg	1	OPC A2B5 CNP-ase		Improved functional recovery Increased angiogenesis

AKI, acute kidney injury; I/R, ischemia/reperfusion; AMI, acute myocardial injury; TBI, traumatic brain injury; SIC, subcortical ischemic stroke; n.d., not defined; SD, Sprague Dawley; SCID, severe combined immunodeficiency; f, female; m, male; y, yes; n, no; EV, extracellular vesicles; i.v., intravenously; i.a., intraabdominal; i.p., intraperitoneal; i.s., intrasplenic; µg, microgram; CE, cell equivalent; ml, milliliter; MV, microvesicle; CD, cluster of differentiation; IL, Interleukin.
